# Visceral Angiosarcoma: A Nationwide Population-Based Study from 2000–2017

**DOI:** 10.3390/cancers17132101

**Published:** 2025-06-23

**Authors:** Lasse Rehné Jensen, Christina Enciso Holm, Johan Tolstrup, Mathias Ørholt, Michael Mørk Petersen, Luit Penninga

**Affiliations:** 1 Department of Surgery and Transplantation, Rigshospitalet, Copenhagen University Hospital, 2100 Copenhagen, Denmark; lasse.rehne.jensen@regionh.dk (L.R.J.); johan.tolstrup@regionh.dk (J.T.); 2 Department of Orthopedic Surgery, Rigshospitalet, University of Copenhagen, 2100 Copenhagen, Denmark; christina.enciso.holm@regionh.dk (C.E.H.); michael.moerk.petersen@regionh.dk (M.M.P.); 3 Department of Plastic Surgery and Burns Treatment, Rigshospitalet, University of Copenhagen, 2100 Copenhagen, Denmark; mathias.oerholt.nielsen@regionh.dk; 4 Institute of Clinical Medicine, University of Copenhagen, 2100 Copenhagen, Denmark; 5 Department of Gastrointestinal Surgery, Aalborg University Hospital, 9000 Aalborg, Denmark

**Keywords:** angiosarcoma, visceral, surgery, nationwide, incidence

## Abstract

**Introduction**: Angiosarcomas arise from vascular or lymphatic endothelial cells and can develop at any site. Visceral angiosarcomas are aggressive high-grade tumors with a high risk of recurrence, metastasis, and poor survival. Nationwide studies with long-term follow-up are limited, but crucial for understanding this malignancy. This study aimed to describe a national cohort of patients with visceral angiosarcomas and estimate long-term survival, local recurrence, and metastases. **Methods**: We included all adult patients in Denmark diagnosed with histologically confirmed visceral angiosarcoma from 2000 to 2017. Data were obtained from the Danish Pathology Register and the Danish Sarcoma Database, both providing nationwide and comprehensive records. Additional information on demographics, comorbidities, symptoms, diagnosis, tumor location, treatment, recurrence, and survival were collected from registries and health records. **Results**: Eighteen patients with visceral angiosarcoma were identified, corresponding to an incidence of one per 5.5 million inhabitants per year. The median age was 56.5 years (IQR: 50–70), and 56% were female. Tumors were most commonly located in the kidney, liver, and thoracic wall. Metastases were present at diagnosis in 17% and developed later in 50%. Surgery was performed in 61%, with R0 resection in 55%. Median overall survival was 249 days (IQR: 121–858), and the 5-year survival rate was 11%. Only one patient (6%) remained alive at long-term follow-up. **Conclusions**: This Danish nationwide study confirms that visceral angiosarcomas are rare, highly aggressive tumors with a poor prognosis, consistent with international findings. Despite the small cohort, the disease demonstrated significant heterogeneity in anatomical location, metastatic pattern, and treatment approaches.

## Simple Summary

Visceral angiosarcomas are rare, aggressive cancers with poor survival rates and limited treatment options. Because they are so uncommon, there is little long-term data available to guide clinical decision-making. This study aimed to provide a nationwide overview of patients diagnosed with visceral angiosarcoma in Denmark, analyzing their characteristics, treatments, and outcomes. We identified 18 cases over 17 years, highlighting the disease’s rarity. Most tumors were located in the kidney, liver, or thoracic wall, and over half of the patients developed metastases. Despite surgical treatment in some cases, overall survival remained low, with only one long-term survivor. These findings confirm the aggressive nature of visceral angiosarcoma and underline the urgent need for better diagnostic and treatment strategies. This study contributes valuable national data to improve understanding of this disease and support future research.

## 1. Introduction

Angiosarcoma is a rare and highly aggressive malignant tumor originating from endothelial cells of blood vessels and, less frequently, from lymphatic endothelial cells [1]. It accounts for less than 2% of all soft tissue sarcomas and primarily affects adult and elderly patients, with its incidence thought to be increasing [2]. Angiosarcomas are a clinically and genetically diverse malignancy that can develop in various anatomical sites and is associated with poor overall survival [2,3,4].

Most angiosarcomas arise de novo from mesenchymal tissue. Secondary angiosarcomas are associated with well-documented external exposures, including radiation therapy, chronic lymphoedema (Stewart–Treves syndrome), and several familial syndromes, such as Maffucci syndrome, neurofibromatosis, and Klippel–Trenaunay syndrome [1,5]. Angiosarcomas are commonly classified by location, with cutaneous tumors in the head and neck region being the most frequent subtype [6]. It has also been reported that these tumors can predominantly be deep-seated and located in the extremities [7]. In contrast, visceral angiosarcomas are much rarer and present significant diagnostic and therapeutic challenges due to their anatomical complexity and late presentation [8,9].

Due to the rarity and complexity of angiosarcomas, there is limited high-quality evidence to guide treatment. Most existing studies are based on small institutional cohorts with inconsistent reporting, which has led to variable findings [10,11,12]. Wide-margin surgical resection is the preferred treatment for localized disease. Adjuvant radiotherapy may reduce local recurrence, though its effect on overall survival remains unclear due to conflicting evidence. The role of chemotherapy remains controversial, with no consensus on the optimal agents or timing; neoadjuvant or adjuvant chemotherapy is not standard practice [1,9]. Multimodal therapy, combining surgery with radiation and/or chemotherapy, is generally recommended for high-grade tumors, positive margins, or metastatic disease [13].

Given these challenges, there is a critical need for more comprehensive and systematic data to better understand visceral angiosarcoma’s natural history, treatment outcomes, and prognostic factors. Furthermore, future research should focus on improving diagnostic methods, evaluating novel systemic therapies, and integrating patient-centered outcomes such as quality of life.

The aim of our study was (1) to describe a consecutive national cohort of patients diagnosed with visceral angiosarcoma (2) and to estimate long-term overall survival, risk of local recurrence, and metastases.

## 2. Materials and Methods

The study was reported according to the Strengthening the Reporting of Observational Studies in Epidemiology (STROBE) Statement: Guidelines For Reporting Observational Studies [14]. The study was approved by the Danish Center for Data Registration (P-2022-92 and R-22017531). Patient consent was not required according to Danish law.

### 2.1. Data Source

In Denmark, nationwide data on sarcoma patients is available through the Danish Sarcoma Registry (DSR) [15], a prospectively maintained database established on 1 January 2009. Patients from 2000 to 2008 were later added via validation with the Danish Cancer Registry and the Danish National Pathology Registry [16]. The DSR includes information on patient demographics, tumor characteristics, diagnostics, treatment details, local recurrence, metastases, comorbidities, and mortality. Additionally, the Danish National Pathology Registry (DNPR) collects pathology reports and diagnoses from all Danish pathology departments, covering nearly 100% of cases due to mandatory reporting from both public and private healthcare providers [16]. The Danish Civil Registry ensures no loss of patient survival follow-up [17].

### 2.2. Patient Population

Through Danish registries, patients diagnosed with primary visceral angiosarcoma were identified using SNOMED codes (M9120x, M9170x, and M9130x) from 2000 to 2017. Danish guidelines mandate that all suspected sarcomas undergo review by a specialized pathologist at a national referral center. Due to the universal healthcare system, all sarcoma patients receive government-funded treatment at one of the two specialized centers by sarcoma surgeons or oncologists, with histological evaluation by board-certified sarcoma pathologists. We obtained data on age, sex, comorbidity, symptoms, diagnosis, location, treatment, recurrence, and survival from the registries and health journals.

### 2.3. Outcomes

The primary outcome was the nationwide incidence rate and the 5-year overall survival (OS). The patients were followed from the date of pathology-confirmed diagnosis until death from any cause or until the end of follow-up (1 January 2023). Secondary outcomes included recurrence-free survival (RFS) and cumulative incidence of local recurrence and metastases. Time to recurrence or metastasis was defined as the interval from diagnosis to the first pathology-confirmed event. The presence of local recurrence or metastases was confirmed through pathological verification, indicating tumor regrowth at the primary or distant site.

### 2.4. Statistical Analyses

Categorical data are presented as counts and percentages, and continuous data are presented as medians with interquartile ranges (IQRs). Overall survival, recurrence-free survival, and metastasis-free survival were estimated using the Kaplan–Meier method. Statistical analysis and plots were performed using R v4.2.2 (R Foundation, Vienna, Austria) software.

## 3. Results

### 3.1. Patient Characteristics

From 2000 to 2017, a total of 172 angiosarcomas were identified across all locations, of which 18 (10%) visceral cases were included in this study corresponding to an incidence of 1 patient per 5.5 million inhabitants per year. The majority of patients were female (56%), and the median age was 56.5 years (IQR: 50–70). Primary tumor location included the kidney (including two donor kidneys) (17%), liver (17%), thoracic wall/soft tissue (17%), followed by lung (11%), spleen (6%), jejunum (6%), caecum (6%), rectum (6%), retroperitoneum/abdomen (6%), aorta (6%), and atrium (6%). Three (17%) patients had metastases at diagnosis, and 50% had metastases later in the course of the disease. Metastases were located in the lung (11%), liver (11%) pleura (6%), gallbladder (6%), retroperitoneum (6%), adrenal gland (6%), and thyroid (6%). The median tumor size was 5 cm (IQR: 3.2–8). One patient (subject 15) had previously received radiotherapy for rectal cancer, and the angiosarcoma was considered a secondary malignancy as a result. Detailed overview is available in Table 1.

### 3.2. Treatment

Treatment included surgery in patients with local disease without metastases (61%), with 64% achieving R0 resection, as well as radiotherapy (33%) and chemotherapy (50%). Radiotherapy was administered with palliative intent in 28% of cases and as adjuvant treatment in 6%, while chemotherapy was used palliatively in 50% (Table 2). Adjuvant radiotherapy was administered for tumors in the kidney and liver, while palliative radiotherapy was used for tumors in the thorax, rectum, and aorta.

### 3.3. Recurrence and Overall Survival

**Figure 1:**
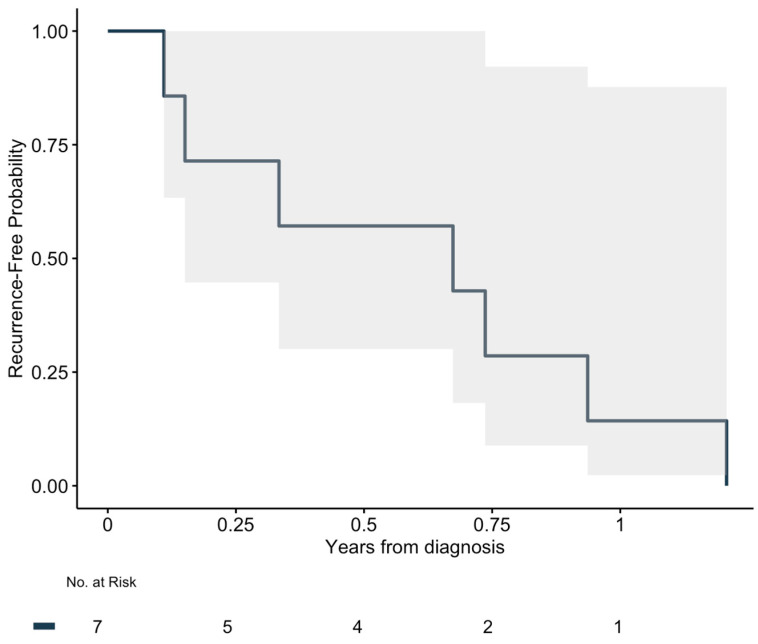
Kaplan–Meier for recurrence.

**Figure 2:**
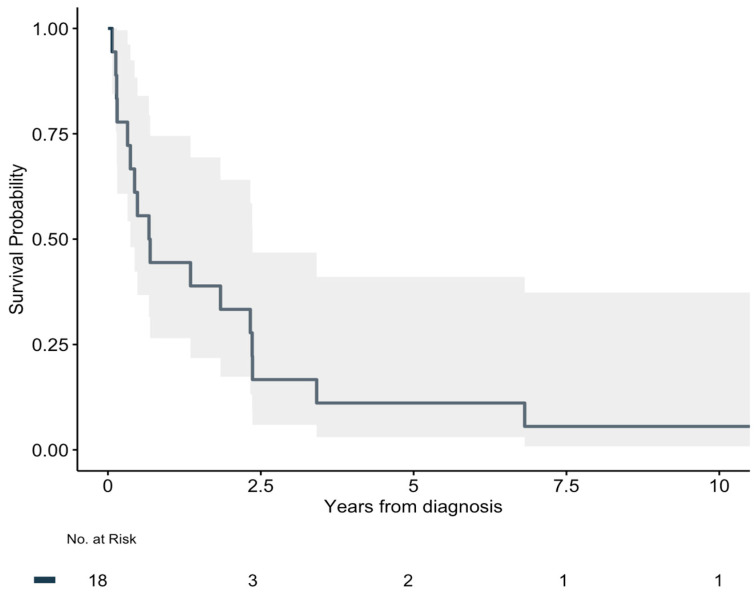
Kaplan–Meier for overall survival.

All patients undergoing R0 resection developed local recurrence or metastases. The median RFS for patients with R0 resection was 246 days (IQR: 89–306) (Figure 1). For the patients with metastases, four (50%) underwent surgery for these. The median OS for all patients was 249 days (IQR: 121–858). The full overview is presented in Table 2. The 5-year overall survival rate was 11% (Figure 2). One (6%) patient was alive at follow-up after 11 years.

## 4. Discussion

This study presents the nationwide characteristics and outcomes of patients with visceral angiosarcoma in Denmark from 2000 to 2017, with complete follow-up. The national incidence was 1 patient per 5.5 million inhabitants per year. Visceral angiosarcomas offered a dismal prognosis, with a 5-year overall survival rate of only 11%. The population showed heterogeneity regarding tumor location, metastases, and treatment. Because of the small cohort, our data do not allow further analysis of risk factors and prognostic factors.

Previous studies report a 5-year survival rate of 30–40% for angiosarcoma, with overall survival ranging from 6 to 16 months [9]. Visceral angiosarcomas have significantly worse outcomes than cutaneous cases, which account for two-thirds of all diagnoses [18]. In this study, the 5-year survival rate was 11%, with a median survival of 8.2 months, which, although comparable, is longer than the 3.8-month median survival reported in a 2022 U.S. nationwide study of 893 visceral angiosarcoma patients [4]. Similarly, a study focusing on 216 patients with primary hepatic angiosarcoma reported a median survival of 6 months and emphasized surgery as the primary treatment strategy [19].

Metastatic disease is associated with an even poorer prognosis and reduced overall survival [20]. Other negative prognostic factors include large tumor size, older age at diagnosis, high-grade tumors, involvement of critical anatomical structures, and lack of R0 resection [4,21,22,23]. Several of these factors were common in our cohort, with a median tumor size of 5 cm, metastases present in 50% of patients, high-grade tumors in all but one case (of the available), and R0 resection achieved in 64% of patients undergoing surgery. However, two patients had very short recurrence-free survival (40 and 55 days), which could be due to undetected metastases at the time of surgery.

The causes of cutaneous angiosarcoma are well-documented, with key risk factors including prior radiation exposure, chronic lymphedema, and benign vascular lesions [12,24,25]. In contrast, the origins of visceral angiosarcoma are less clear, with most cases lacking a specific cause [9,26]. Identified risk factors include radiation and exposure to environmental carcinogens such as vinyl chloride, arsenic, and thorium dioxide [9,27]. In our study, only one patient, previously treated for rectal cancer, had undergone radiation therapy. The cause of the remaining cases was unclear. Two patients in this study developed angiosarcomas after renal transplantation in the transplanted kidney, a rare occurrence previously described in the literature. It has been described both shortly after transplantation and several years later [28]. The two patients in our study received organs from the same deceased donor, whose organs were transplanted into multiple recipients later diagnosed with angiosarcoma. This suggests that the source of the angiosarcomas may have been undiagnosed disseminated angiosarcoma in the donor. In both patients, the transplanted kidneys were surgically removed. One patient passed away 52 days after surgery, while the other remained alive at follow-up after undergoing three additional surgeries for metastases and receiving adjuvant radiotherapy.

Terminal bleeding is a recognized severe complication in patients with angiosarcoma and can occur due to hemorrhagic events from various tumor localizations. Examples include hemothorax resulting from pulmonary metastases or pleural dissemination, gastrointestinal bleeding, and spontaneous hemorrhage from liver or soft tissue lesions [29,30,31,32]. These acute bleeding events may be life-threatening and contribute significantly to morbidity and mortality. Unfortunately, detailed data on bleeding complications were not available in our cohort, preventing further analysis of their incidence and impact. Nonetheless, awareness of such hemorrhagic risks is crucial for clinical management and highlights the aggressive nature of angiosarcoma.

Limited evidence exists on optimal treatment for angiosarcomas, but multimodal treatment, including surgery, radiotherapy, and chemotherapy, is often advised and applied. Management depends on whether the condition is non-metastatic or metastatic. For non-metastatic disease, the goal is complete surgical resection (R0 resection), as R1 and R2 resections are associated with a worse prognosis [33,34,35], which is also the case specifically for visceral angiosarcomas [36]. However, for visceral angiosarcoma, even localized disease is associated with high recurrence risk and mortality. The management of patients in this study with a small cohort size was highly heterogeneous. This was likely due to the rarity of the disease and the inherent variability in its presentation, including differences in anatomical location, disease progression, and the patient’s overall condition, which influenced their ability to receive various treatments. In our population, 61% underwent surgery, but only 55% achieved an R0 resection. All patients had recurrence, either local or metastasized, despite of R0 resection. For both non-metastatic and metastatic cases, other treatment modalities can be used. Chemotherapy regimens based on anthracyclines and taxanes are commonly chosen as first-line treatments in patients with recurrent or irresectable disease; however, the response rate is often limited [3,23,37]. Other targeted therapies, such as several anti-VEGF agents, have been tested, but also failed to show significantly beneficial effects [35]. Recently, immunotherapy has been studied as a treatment for angiosarcomas. In one case series, the authors found that immune checkpoint inhibitors, such as pembrolizumab and axitinib, showed promising results for treating angiosarcoma. Of seven patients, 71% had partial responses, with one achieving complete response [38]. No grade 2 or higher toxicities were observed. Another phase II trial on ipilimumab and nivolumab in metastatic angiosarcoma reported a 25% overall response rate, particularly in patients with scalp or facial tumors. While some adverse events occurred, no grade 5 toxicities were observed [39]. Radiotherapy, while not the first-line treatment for angiosarcoma, has shown benefits, especially for inoperable cases (palliative) and in reducing recurrence post-surgery (adjuvant). High-dose radiation (>70 Gy) has been found to improve local control and overall survival [1]. However, the optimal approach remains unclear, with combined therapy (surgery plus adjuvant radiotherapy) yielding better outcomes than surgery alone. Factors like treatment dose and technique influence efficacy, but further studies are needed to determine the best radiotherapy strategies for angiosarcoma, particularly for large or unresectable tumors [9]. Further investigation of these therapies in angiosarcoma is needed, as the optimal treatment strategy, particularly for visceral angiosarcomas, remains unclear. This is also reflected in our study, where no clear patterns emerged in the choice of radiotherapy and chemotherapy.

Given the aggressive nature of angiosarcoma and its poor prognosis, balancing radical treatment with quality of life (QoL) is essential. Although our study lacks QoL data, a recent systematic review by McDonough et al. highlights the significant physical, emotional, and psychosocial challenges faced by sarcoma patients, resulting in a high symptom burden and impaired QoL [40]. Similarly, Temel et al. showed that early palliative care improved both QoL and survival in metastatic non-small-cell lung cancer—also a disease with poor prognosis [41]. These findings emphasize the importance of integrating supportive care and considering patient preferences in treatment decisions. There is a clear need for prospective studies focused on QoL and patient-centered outcomes in angiosarcoma, including visceral cases, to better guide individualized management strategies.

The major strength of this study lies in the use of nationwide registries, ensuring complete inclusion and long-term follow-up of all patients diagnosed with angiosarcoma over an 18-year period. The consecutive cohort reduces the risk of selection bias. Limitations include the small cohort, which makes it unfeasible to perform further statistical analyses and limits the generalizability of the findings. Despite the use of standardized diagnostic criteria and analysis by all pathologists in Denmark, misclassification and information bias remain potential risks in registry-based studies. As noted, the incidence of metastasis is likely underestimated, as only patients with pathologically confirmed metastasis were included in the analysis. However, the individual patient data provided in this study can be valuable for clinicians and researchers beyond our department.

## 5. Conclusions

This Danish nationwide population-based study confirmed that visceral angiosarcomas are rare, aggressive tumors with a poor prognosis, consistent with findings from other countries. Despite the small cohort, the disease showed considerable heterogeneity in anatomical location, metastatic patterns, and treatment approaches. Further prospective studies are needed to establish optimal treatment strategies. By adding individual patient data, this study contributes valuable insights to the global literature on this rare disease.

## Figures and Tables

**Table 1:** Patient and angiosarcoma characteristics.

Subject	Sex Female (%)	Age, Years Median (IQR)	Location of Primary Tumor	Tumor Size, cm Median (IQR)	Metastases at Diagnosis Yes (%)	Metastases, Later Yes (%)	Metastases, Location
All	56%	56.5 years (50–70)		5 cm (3.2–8)	17%	50%	
1.	Male	75	Internal thoracic wall	10	No	No	
2.	Female	22	Internal thoracic wall or pleura	1.7	No	No	-
3.	Male	73	Thoracic soft tissue	N/A	No	No	-
4.	Male	36	Lung	6	No	Yes	Adrenal gland
5.	Female	70	Lung	5	No	No	-
6.	Male	44	Deceased donor kidney	2.1	No	Yes	Retroperitoneum
7.	Male	40	Deceased donor kidney	22	Yes	Yes	Lung
8.	Male	50	Kidney	5	No	No	-
9.	Female	51	Liver	10	Yes	Yes	Gallbladder
10.	Female	68	Liver	3.5	No	No	-
11.	Female	81	Liver	5.2	No	No	-
12.	Female	69	Spleen	N/A	No	Yes	Liver
13.	Female	76	Jejenum	6.7	No	Yes	Liver
14.	Male	57	Caecum	0.8	No	Yes	Pleura
15.	Female	59	Rectum	3.1	No	Yes	Thyroid
16.	Female	53	Retroperitoneum/abdomen	9.3	No	No	-
17.	Female	56	Aorta	3.2	No	Yes	-
18.	Male	55	Atrium	N/A	Yes	Yes	Lung

N/A: Not available.

**Table 2:** Treatment and outcomes.

Subject	Location of Primary Tumor	Surgery Yes (%)	R0 Yes (%)	RT Adjuv. Yes (%)	RT Pall. Yes (%)	Chemo Adjuv Yes (%)	Chemo Pall. Yes (%)	Recurrence or Mets. for R0 Yes (%)	Recurrence Free Survival, Days Median (IQR)	Surgery for Mets. Yes (%)	Death Yes (%)	Overall Survival, Days Median (IQR)
All		61%	64%	6%	28%	0%	50%	100% of R0	246 days (89–306)	50% of mets.	94%	249 days (121–858)
1. Male, 75 years	Internal thoracic wall	No	-	No	No	No	No	-	-	-	Yes	47
2. Female, 22 years	Internal thoracic wall or pleura	Yes	No	No	No	No	Yes	-	-	-	Yes	252
3. Male, 73 years	Thoracic soft tissue	No	-	No	Yes	No	No	-	-	-	Yes	117
4. Male, 36 years	Lung	Yes	Yes	No	Yes	No	Yes	Yes	269	Yes, two	Yes	1246
5. Female, 70 years	Lung	Yes	Yes	No	No	No	Yes	Yes	40	-	Yes	55
6. Male, 44 years	Deceased donor kidney	Yes	No	Yes	No	No	Yes	-	-	Yes, three	No	4027
7. Male, 40 years	Deceased donor kidney	Yes	No	No	No	No	No	-	-	No	Yes	52
8. Male, 50 years	Kidney	Yes	No	No	No	No	Yes	-	-	-	Yes	176
9. Female 51 years	Liver	Yes	Yes	No	Yes	No	No	Yes	441	Yes, one	Yes	2488
10. Female 68 years	Liver	No	-	No	No	No	Yes	-	-	-	Yes	245
11. Female, 81 years	Liver	No	-	No	No	No	No	-	-	-	Yes	493
12. Female, 69 years	Spleen	Yes	Yes	No	No	No	Yes	Yes	246	No	Yes	863
13. Female, 76 years	Jejenum	Yes	Yes	No	No	No	No	Yes	342	-	Yes	672
14. Male, 57 years	Caecum	Yes	Yes	No	No	No	No	Yes	122	No	Yes	134
15. Female, 59 years	Rectum	No	-	No	Yes	No	Yes	-	-	Yes, one	Yes	861
16. Female, 53 years	Retroperitoneum/abdomen	No	-	No	No	No	No	-	-	-	Yes	25
17. Female, 56 years	Aorta	Yes	Yes	No	Yes	No	Yes	Yes	55	-	Yes	850
18. Male, 55 years	Atrium	No	-	N/A	N/A	N/A	N/A	-	-	No	Yes	159

N/A: Not available.

## Data Availability

The data presented in this study have restrictions due to patient confidentiality and are, therefore, not publicly available. However, they can be made available on reasonable request from the corresponding author.

## Abbreviations

The following abbreviations are used in this manuscript:

STROBE	Strengthening the Reporting of Observational Studies in Epidemiology
DSR	Danish Sarcoma Registry
DNPR	Danish National Pathology Registry
OS	Overall Survival
RFS	Recurrence-Free Survival
IQR	Interquartile Range
SD	Standard Deviation
SNOMED	Systematized Nomenclature of Medicine
IBM	International Business Machines
SPSS	Statistical Package for the Social Sciences
VEGF	Vascular Endothelial Growth Factor
QoL	Quality of Life
